# Not recommended fixed-dose antibiotic combinations in low- and middle-income countries – the example of Tanzania

**DOI:** 10.1186/s13756-023-01238-8

**Published:** 2023-04-19

**Authors:** Klaske Vliegenthart-Jongbloed, Jan Jacobs

**Affiliations:** 1 Haydom Lutheran Hospital, Haydom, United Republic of Tanzania; 2 Department of Medical Microbiology and Infectious Diseases, Erasmus MC, Rotterdam, the Netherlands; 3 Department of Clinical Sciences, Institute of Tropical Medicine Antwerp, Antwerp, Belgium; 4 Department of Microbiology, Immunology and Transplantation, KU Leuven, Leuven, Belgium

**Keywords:** Fixed-dose combinations (FDC), Antibiotics, WHO AWaRe classification, Low- and middle-income countries, Antimicrobial stewardship, Prescriber, Regulation

## Abstract

**Background:**

Fixed-dose combinations (FDC) are medicine formulations that combine two or more ingredients in fixed ratios in a single dose form. Although advantageous in tuberculosis and malaria (efficacy, adherence, protection against resistance), only a few antibiotic FDC (FDC-AB) have been developed along full microbiological, pharmacological and clinical validation and safety studies. The World Health Organization (WHO) database of Access, Watch and Reserve (AWaRe) antibiotics contains, since 2021, a list of “Not Recommended” FDC-AB (n = 103) which are rejected for use in clinical practice.

**Body:**

The share of non-recommended FDC-AB in global antimicrobial use (2000–2015) was < 3% but substantially higher in middle income countries. The share increases over time, but recent data particular concerning sub-Saharan Africa are rare. Along three non-recommended FDC-AB listed in the Tanzanian National Essential Medicine List (ampicillin-cloxacillin, flucloxacillin-amoxicillin and ceftriaxone-sulbactam) we discuss the concerns and reasons behind use of these products. Non-recommended FDC-AB have poor rationale (ratios of both ingredients), lack evidence of efficacy (pharmacological, microbiological and clinical), have difficulties in dosing (underdosing of the single ingredients, absence of pediatric dosing) and risks of safety (additive toxicity). They are expected to fuel antimicrobial resistance (unnecessary broad spectrum coverage) and are incompatible with antimicrobial stewardship. The specific context of low- and middle-income countries contributes to their increased use: at the side of prescriber and supplier are the lack of diagnostics, poor training in antibiotic prescribing, patients’ preferences, role-model of senior prescribers and pharmaceutical promotion. International market mechanisms include economic motivation for development, branding and promotion, poor access to the single antibiotic forms and weak national regulatory capacity.

**Conclusion and implications:**

There is an urgent need for monitoring consumption of non-recommended FDC-AB in low- and middle-income countries, particular in Sub-Saharan Africa. A multinational and multisectoral antimicrobial stewardship strategy is needed in order to abolish the use of non-recommended FDC-AB.

**Supplementary Information:**

The online version contains supplementary material available at 10.1186/s13756-023-01238-8.

## Background

Fixed-dose combinations (FDC) are medicine formulations that combine two or more active ingredients in fixed ratios in a single dose form [1]. FDC antimicrobials have their place in the treatment of infectious diseases such as HIV, malaria and tuberculosis, providing advantages over the single ingredients, such as increased efficacy, improved adherence and protection against emergency of resistance [2, 3]. Combining different antibiotics is very common in clinical practice if a broad coverage is required, for example in empirical antibiotic treatment (i.e. before results of microbiological analysis are known) or in case of mixed flora infections (e.g. intraabdominal infections). Usually separate drugs are administered based on personalized needs, while waiting for recovery and microbiological results in order to switch to a smaller spectrum once possible [4, 5].

So why combine antibiotics in an antibiotic fixed-dose combination (FDC-AB), in which the separate components cannot be adjusted? The main rationale is to achieve synergy in order to improve efficacy, such as in the case of the congruous combination of trimethoprim-sulfamethoxazole [6]. The combination of a beta-lactam antibiotic with beta-lactamase inhibitor (BL-BLI) is a so called syncretic combination [6, 7]: adding clavulanic acid (which has only minor intrinsic antibiotic effect) to amoxicillin restores susceptibility of several beta-lactamase producing bacteria. Although reducing the risk of emerging resistance during treatment is an essential argument in favor of FDC in malaria and tuberculosis treatment, synergistic combinations of antibiotics can counterintuitively drive more rapid evolution of resistance than individual antibiotics [6, 7]. Moreover, antagonism is common in antibiotic combinations, such as one antibiotic which inhibits the cell death mechanism of another antibiotic, for example if combining a bacteriostatic inhibitor of protein synthesis with a bacteriocidic beta-lactam antibiotic (e.g. doxycycline and ceftriaxone) [6, 7].

Because the effect of a FDC-AB is not equal to the sum of its parts, the formulation must meet specific criteria, related to medical and pharmacological rationale as well as to safety and therapeutic efficacy [8, 9]. Table 1 lists the scientific requirements according to European Medicines Agency (EMA) [8]. For an excellent overview of the pathway of non-clinical microbiology studies, animal experiments and clinical phase I – III studies, we refer to reference *Palwe et al.* [10]. Only few FDC-AB have met these scientific and regulatory requirements. The World Health Organization (WHO) database of Access, Watch and Reserve (AWaRe) antibiotics contains several approved antibiotic FDCs, which we listed in Supplementary Table 1, together with their approval status by the United States Food and Drug Administration (US FDA) and the EMA and whether or not they are listed in the WHO Model List of Essential Medicines (WHO EML) [11–14].

**Table 1:** Basic scientific requirements for a fixed-dose combination [8]

1. Justification of the **pharmacological and medical rationale** for the combination
2. Establishment of the **evidence base** for the:
a. relevant contribution of all active substances to the desired therapeutic effect (efficacy and/or safety)
b. positive benefit-risk for the combination in the targeted indication
3. Demonstration that the **evidence presented is relevant to the fixed combination medicinal product** for which the application is made

Adapted from: EMA. Guideline on clinical development of fixed combination medicinal products 2017 (8)

Since 2021 WHO added to the AWaRe classification an additional category of “Not Recommended” antibiotics [11, 12], further referred to as ‘non-recommended FDC-AB’. In an brief explanatory note, WHO states that these FDC-AB are not evidence-based nor recommended by international guidelines [15]. Moreover, concerns are expressed about efficacy, safety, dosing, and the emergence of antimicrobial resistance (AMR) [16, 17]. Despite these potential harms, non-recommended FDC-AB are still frequently used, particularly in low- and middle-income countries [16, 17].

To increase awareness and knowledge about non-recommended FDC-AB, we presently discuss three FDC antibiotics present on the Model List of Essential Medicines of Tanzania. We explain the lack of evidence (microbiological, pharmacological and clinical) for the use of these FDC antibiotics, discuss the concerns about safety and enhancing AMR and highlight their incompatibility with antibiotic stewardship. We further explore the extent and reasons behind their increased use in low-and middle-income countries.

### Antibiotic resistance and antibiotic use in Tanzania

Recently (2020), the United Republic of Tanzania moved from being a low-income country to a lower-middle-income country [18]. Tanzania is one of the four African countries owning a Medicines and Medical Devices Authority (TMDA) on maturity level 3, judging both medicines as well as vaccines [19]. Mainland Tanzania has a National Essential Medicine List (NEMLIT), which is currently in its sixth edition (2021) with 70% compatibility with the WHO EML [20]. The NEMLIT is published combined with the Standard Treatment Guidelines (Tanzanian STG) which not only addresses antibiotic treatment, but treatment in general for both adults as well as children [21]. A recent point prevalence survey on antibiotic use in six referral hospitals in Tanzania highlighted that 84% of prescriptions was in accordance with national guidelines [22].

Access to microbiological laboratories in Tanzania is limited: according to the aforementioned study in referral hospitals in Tanzania, only 2 out of 591 patients were prescribed antibiotics based on antimicrobial susceptibility testing results [22]. Consequently, there is a lack of national or local surveillance data on bacterial pathogens and their AMR patterns. Although Tanzania is building the way towards AMR surveillance, there were no aggregated data submitted yet to the 2021 WHO Global AMR and Use Surveillance System (GLASS) report [23].

Although the overall consumption of antimicrobials in Tanzania is high (80 Defined Daily Doses (DDD) per 1000 inhabitants per day as compared to 18,4 in the EU in 2018 and 9,1 in China in 2017), a decrease was observed between 2017 and 2019, from 136 to 51 DDD per 1000 inhabitants per day. [24]. Tanzania however excels in the use of antibiotics from the Access class (> 90%), which is considerably higher than the minimum of 60% targeted by WHO [15, 24].

### FDC antibiotics in the Tanzanian Essential Medicines List and Standard Treatment Guidelines

In Tanzania, a significant portion of antibiotic use involves the usage of non-recommended FDC-AB. According to a report on antibiotic consumption in Tanzania from 2017 to 2019, the top 10 consumed antimicrobials included combinations of norfloxacin-tinidazole and combinations of penicillins. These combinations accounted for 2.27 and 1.33 DDDs per 1000 inhabitants per day, respectively, with the private sector having the highest share [24].

The FDC ampicillin-cloxacillin accounted for 17,5% of antibiotic prescriptions in 6 surveyed Tanzanian hospitals [22]. Three non-recommended FDC-AB have been adopted by NEMLIT and Tanzanian STG (2021): ampicillin-cloxacillin, flucloxacillin-amoxicillin and ceftriaxone-sulbactam [21]. Tables 2 and 3 show their formulations and recommended indications for use respectively. Together they are recommended as first line treatment options for more than 28 indications, including three prophylaxis indications with an additional indication for second line treatment.

**Table 2:** Fixed-dose combination antibiotics on the Tanzanian 2021 National Essential Medicines List (NEMLIT) [22]

Drug combination	Fixed dose combinations^1^	Tanzania EML AWaRe classification and level of allowed prescription^2^	WHO AWaRe classification^3^
**Not listed on the WHO essential medicine list (EML)**			
ampicillin-cloxacillin	Caps 250/250 mg Inj 250/250 mg	Access (B)	**Not Recommended**
flucloxacillin-amoxicillin	Table 250/250 mg	Access (C)	**Not Recommended**
ceftriaxone-sulbactam	Inj 1000/500 mg	Watch (D)	**Not Recommended**
ampicillin-sulbactam	Inj 1000/500 mg Inj 2000/1000 mg Inj 10.000/5000 mg	Watch (C)	**Access** (but not listed on the WHO EML)
**Listed on the WHO essential medicine list (EML)**			
amoxicillin-clavulanic acid	Susp 125/31,25 mg Susp 250/62,5 mg Table 500/125 mg	Access (B)	Access (EML)
	Inj 500/100 mg	Access (C)	
piperacillin-tazobactam	Inj 2000/250 mg Inj 4000/500 mg	Watch (S)	Watch (EML)
sulfamethoxazole- trimethoprim	Susp 200/40 mg Table 400/80 mg	Watch (A)	Access (EML)

Note: Combinations of antimicrobials intended for treatment or prophylaxis of tuberculosis, malaria or viral infections are not included in this overview^1^. Abbreviations : Caps = capsule, Inj = powder for injection, Susp = power for suspension (dose per 5 mL), Tab = tablet. Tanzania AWaRe classification and level of prescription^2^: assessed using the Tanzanian 2021 guidelines (21). Level of prescription indicates if there is a restriction with regard to service provision or professional expertise. It indicates the lowest level for which prescription is allowed. B: health centre/clinical officer, C: district hospital/assistant medical officer, D: regional referral hospital/ medical officer, S: tertiary hospital/specialist. WHO EML 2021 AWaRe classification^3^: assessed using the online database (12) (https://aware.essentialmeds.org/list) and the Excel database document (11) (https://www.who.int/publications/i/item/2021-aware-classification)

**Table 3:** Listed indications for Not Recommended fixed-dose combination (FDC) antibiotics on the Tanzanian 2021 National Essential Medicines List (NEMLIT) [21]

Ampicillin + Cloxacillin (combination of penicillins)
Mild community acquired pneumonia *Staphylococcus aureus* pneumonia Lung abscess (with metronidazole) Orbital cellulitis (with gentamicin and metronidazole and vancomycin) Circumscript otitis externa Herpes Zoster Opticus (with acyclovir) Hematoma or laceration of the pinna (outer ear, assumed prophylaxis) Nasal septal abscess and hematoma Infection of cyst (thyroglossal duct, dermoid or branchial cleft) (with cephalexin) Endodontic treatment for dental caries (with metronidazole) Acute osteomyelitis of the jaw (with metronidazole) Facial bone injuries (prophylaxis if suspected contamination of extensive damage)
**Flucloxacillin + Amoxicillin (combination of penicillins)**
Mastitis Erysipelas/Cellulitis Abscess in face or immunosuppressed patient Uncomplicated cystitis
**Ceftriaxone + Sulbactam (combination of BL + BLI)**
Bacterial meningitis Brain abscess (with metronidazole) Brain abscess with *S. aureus* (as an addition to vancomycin) Pediatric hydrocephalus (surgical prophylaxis in children) Pediatric CSF shunt infections (with metronidazole) Severe community acquired pneumonia Hospital acquired pneumonia Lung abscess Septic abortion (second line treatment if amoxicillin-clavulanic acid fails, with metronidazole) Chorioamnionitis (with metronidazole) Puerperal sepsis (with metronidazole) Surgical prophylaxis in caesarean section (with metronidazole) Urosepsis (with gentamicin)
Abbreviations: BL + BLI = β-lactam antibiotic in combination with a β-lactamase inhibitor, PO = per oral, IV = intravenous

### The penicillin combinations: ampicillin–cloxacillin and flucloxacillin-amoxicillin

The penicillin combinations ampicillin-cloxacillin and flucloxacillin-amoxicillin are both FDC antibiotics consisting of an aminopenicillin (ampicillin respectively amoxicillin) combined with a penicillinase-resistant anti-staphylococcal antibiotic (cloxacillin respectively flucloxacillin).

There is no microbiological rationale for combining these two types of penicillin. Although both ingredients can target streptococcal infection, there is no advantage in adding anti-staphylococcal action (Table 4). If the cause of infection is *S. aureus*, the aminopenicillin is meaningless. If the infection is caused by (susceptible) Gram-negative bacteria or enterococci, the addition of anti-staphylococcal antibiotics is obsolete. The recommendation to treat uncomplicated cystitis with the FDC flucloxacillin-amoxicillin is not rational as flucloxacillin does not cover uropathogens.

**Table 4:** Characteristics of the individual ingredients of penicillin combinations in Tanzania

Ingredient	Recommended use^1^	Active but Not Recommended^1^	Standard dosage^2^
flucloxacillin or cloxacillin	*S. aureus* (methicillin susceptible) Susceptible coagulase negative staphylococci (CNS)	*Streptococcus pyogenes* (A), *Streptococcus agalactiae* (B) Streptococci group C,F,G, *Streptococcus anginosus* *Streptococcus pneumoniae*	Oral flucloxacillin: 1 gram, 8 hourly Oral cloxacillin: 500 mg, 6 hourly
ampicillin or amoxicillin	*Actinomyces*, Susceptible *E. faecalis* *L. monocytogenes* *N. meningitidis* *P. multocida* Peptostreptococci	*Streptococcus pyogenes* (A), *Streptococcus agalactiae* (B) Streptococci group C,F,G, *Streptococcus anginosus* *Streptococcus pneumoniae* *Clostridium* among others	Oral amoxicillin: 500 mg, 8 hourly Oral ampicillin: 250–500 mg, 6 hourly^1^ (amoxicillin has increased oral absorption compared to ampicillin due to added hydroxyl group^1^)

^1^ Spectrum of activity and recommended use is based the Sanford Guide [68]

^2^ Standard dosage is based on the EUCAST clinical breakpoints and dosing Table (25), except for oral ampicillin which is not listed in EUCAST.

Additionally, there is no pharmacological advantage for the penicillin combinations, as the two ingredients are not synergistic, nor improve each other’s bioavailability. Moreover, the content of per dosage of both ingredients is insufficient to obtain the required dose for effective treatment of targeted infections. As an example, the Tanzanian STG recommend FDC ampicillin-cloxacillin for treatment of *S. aureus* pneumonia (4 gram IV per day) and lung abscess (1,5 − 3 gram IV per day). Because only half of the FDC exerts anti-staphylococcal activity, this results in a cloxacillin daily dose of 2 respectively maximum 1,5 gram IV as opposed to a recommended dose of 4 to 12 gram IV per day depending on severity and location of the infection [25]. Likewise the recommended oral treatment in the Tanzanian STG for osteomyelitis after initial IV treatment is 1,5 gram PO per day, representing 0,75 gram cloxacillin as opposed to the recommended 2–4 gram with the known caveat of poor bone penetration [25, 26]. The recommended daily dose of amoxicillin-flucloxacillin in cellulitis is 2 gram and for mastitis 1 gram, which results in an effective flucloxacillin dose of 1 gram respectively 500 mg. This daily dose is low compared to the standard oral flucloxacillin dose of 3 gram per day according to the European committee on antimicrobial susceptibility testing [25].

Finally, clinical studies showed there is no evidence to support the proof of concept to add a penicillin to flucloxacillin in the treatment of cellulitis [27–29]. Likewise, data about efficacy or toxicity of ampicillin-cloxacillin as an FDC are lacking [30].

### The beta-lactam–beta-lactamase inhibitor combination: ceftriaxone–sulbactam

Ceftriaxone-sulbactam is a BL-BLI combination. In the combination ceftriaxone-sulbactam a clinician would presume sulbactam prevents the breakdown of ceftriaxone by relevant beta-lactamases, in analogy to amoxicillin-clavulanic acid or piperacillin-tazobactam. Sulbactam however shows particular activity against class A beta-lactamases only and has no activity against class B, C or D beta-lactamases [31]. A microbiological study in contemporary Indian clinical isolates showed that ceftriaxone Minimal Inhibitory Concentration (MIC) values in the presence of sulbactam remained high for ESBL/class C- as well as for Metallo Beta-Lactamase-expressing (Class B) *Enterobacterales* [10].

Further, there is no pharmacological rationale behind the fixed dose ratio of ceftriaxone-sulbactam [10]. Different BL-BLI combination pairs show different pharmacokinetic interactions, and consequently the required ratios vary significantly. For example piperacillin-tazobactam (Watch antibiotic) is dosed in a 8:1 ratio based on dose-determining studies, whereas the 2:1 dose ratio in ceftriaxone-sulbactam is copied from other non-recommended FDC-AB and not based on pharmacological studies [10].

An additional pharmacological concern is the difference in pharmacokinetics between ceftriaxone and sulbactam. Sulbactam has a serum half-life of only 1 h, resulting in a mismatch with the ceftriaxone dosing frequency which is once or twice daily [32]. In addition, its penetration in inflamed meninges is low and variable [32]. Nevertheless, in the Tanzanian STG, ceftriaxone-sulbactam is recommended -among other indications- for hospital acquired pneumonia, urosepsis, brain abscess and bacterial meningitis [21]. The recommended daily dose of ceftriaxone-sulbactam in meningitis and brain abscess is 3 gram, which results in a ceftriaxone daily dose of only 2 gram as opposed to the recommended 4 gram for intracerebral infections in international literature [25, 33–35]. The recommended daily dose of ceftriaxone-sulbactam in urosepsis is 1 gram, which is not feasible considering a vial contains 1500 mg [21]. Moreover, the internationally recommended daily ceftriaxone dose in urosepsis is 2 gram [25, 35, 36].

Ultimately, a PubMed search on randomized trials concerning ceftriaxone-sulbactam did not result in any studies reporting clinical outcomes.

### Overview of FDC antibiotics listed as “not recommended” by the World Health Organization

Beside the 3 FDC-AB mentioned above, the WHO AWaRe database lists another 100 non-recommended FDC-AB [11]. The complete list consist of either two (n = 74), three (n = 26) or even four (n = 2) or five (n = 1) ingredients. Among the proliferation of antimicrobial combinations, 21 contain a probiotic (e.g. *Lactobacillus* spp.) and 5 contain a non-antimicrobial ingredient (e.g. mucolytic substances like bromhexine). Forty-one (39.8%) are BL-BLI combinations, 16 (15.5%) combine a beta-lactam antibiotic with a beta-lactamase stable penicillin, like ampicillin-cloxacillin. A total of 66 (64.0%) FDC antibiotics contain Watch group antibiotics, including a cephalosporin antibiotic (n = 42/103, 40.8%; mostly oral cephalosporines cefixime and cefpodoxime), fluoroquinolones (n = 16, 15.5%) and macrolides (n = 11, 10.7%, of which azithromycin n = 6). Two products contain the Reserve antibiotic linezolid [11].

Non-recommended FDC-AB are also listed among different groups in the WHO Anatomical Therapeutic Chemical (ATC) classification system, which is issued and maintained by the WHO Collaborating Centre of Drugs Statistics Methodology (WHOCC). This list is however intended for monitoring of drug utilization, and does not express clinical recommendation nor regulatory approval [36].

### Non-recommended FDC antibiotics: concerns about efficacy, dosing and safety

As we illustrated, the non-recommended FDC-AB don’t serve a microbiological purpose, and without pharmacological and clinical studies the desired synergy might as well be an antagonistic relationship which can result in reduced efficacy [8–10, 37].

Two ingredients very often have incompatible half-lives, resulting in an impaired dosing schedule of one of the components [10]. An example is the dosing schedule of azithromycin (once daily) which is incompatible with its FDC partner drug cefpodoxime (twice daily) [37]. A fixed dose impairs titration of individual ingredients [38] which could incite a prescriber to either underdose the essential ingredient or overdose the unnecessary ingredient.

Safety issues related to non-recommended FDC-AB are increased risks of drug interactions [38] or additive adverse drug reactions such as for instance prolonged cardiac QT-interval in case of combination of azithromycin and fluoroquinolones [37]. An additional concern is the frequent absence of dose recommendations for children and patients with renal dysfunction [10].

### Not-recommended FDC antibiotics: concerns about AMR

Non-recommended FDC-AB are expected to induce and fuel AMR. The number of deaths associated with AMR in 2019 was already estimated 4.95 million, of which the largest share took place in the Sub-Sahara African region [39]. Both human antimicrobial overuse as well as misuse are the major drivers for antimicrobial resistance (AMR) [40]. Antimicrobial stewardship is needed to prevent AMR, which can be defined as the optimal selection, dose, and duration of an antimicrobial that results in the best clinical outcome for the treatment or prevention of infection, with minimal toxicity to the patient and minimal impact on subsequent resistance [41, 42].

So why are non-recommended FDC-AB incompatible with antimicrobial stewardship programs?

The above mentioned difficulty of dosing the ingredients may cause a too low dose which is a known risk factor for emergence of AMR [7]. Conversely, the inherent presence of two antibiotic ingredients instead of one will increase the selective pressure of antibiotics and hence conducive to the emergence of resistance in the infecting as well as commensal flora [7]. Moreover, non-recommended FDC-AB may encourage prolonged ‘shotgun’ therapy, which conflicts with the principle of choosing the antibiotic with the smallest spectrum [43]. They also may promote unnecessary antimicrobial treatment e.g. by using the combination a fluoroquinolone with a antiprotozoal antibiotic (such as ofloxacin-ornidazole) as a first-line treatment for diarrhea [37].

### Non-recommended FDC antibiotics are mostly used in low- and middle-income countries

A report by *Klein et al.* assessing antibiotic consumption in 76 countries between 2000 and 2015 showed that non-recommended FDC-AB were used in 20 (26%) countries [16]. Their share among the total antibiotic consumption was less than 3% on the global scale, but substantially higher in Egypt (9.6%), India (7.5%) and Pakistan (4.0%). *Bortone* and co-workers assessed worldwide antibiotic sales data and recorded 119 FDC antibiotics, the vast majority (110/119, 92%) of which were not approved by the US FDA and represented 5.1% of the total antibiotic sales volume [17]. The highest numbers of FDC antibiotics were noted in India, China and Vietnam (80, 25 respectively 19 out of 119). India and Francophone West Africa (aggregated country data) had the highest percentage of antibiotic FDCs not approved by the US FDA (93.8% of total available FDC-AB) [17].

Although the sale of non-recommended FDC-AB seems to be prevalent in low-resource settings, the only low-income countries included in the worldwide data are aggregated in “Francophone West Africa” [17]. Likewise, the two recently published papers on global consumption only featured data from South Africa and Francophone West Africa, representing Sub-Saharan Africa (16–17). As of now, no worldwide data beyond 2015 has been published.

A policy brief in 2022 from the MAAP (Mapping Antimicrobial Resistance and Antimicrobial Use Partnership) project however, showed that 3,4% of the total antibiotic consumption across the 14 African countries – including Tanzania- were non-recommended FDC-AB [69]. Non-recommended FDC-AB were earlier observed to be prescribed in Uganda, Tanzania and Ghana [44]. In Tanzania, the non-recommended ‘penicillin combinations’ ampicillin-cloxacillin and flucloxacillin-amoxicillin are among the top ten of national antimicrobial consumption [24] and comprised of 18% of the total prescriptions in a point prevalence survey in six referral hospitals [22]. In Uganda, ampicillin-cloxacillin was in 2019 still among the top 5 of consumed antibiotics in Uganda [45].

The use of FDC antibiotics is increasing: sales data in India between 2007 and 2012 showed that the 26% increase was especially due to the growth in sales of FDC antibiotics, which rose by 38% and mainly consisted of non-recommended FDC-AB. Among 118 FDC antibiotics, 64% had no record of approval by India’s national regulator and only 4% were approved by either the UK or USA regulators [46]. In 2020, FDC accounted for 37% of antibiotic sales in India as opposed to single drugs, with non-recommended FDC-AB accounting for 41,5% [47].

### Factors fueling the use of FDC antibiotics in low- and middle income countries

Several reasons can be identified to drive the prescription of non-recommended FDC antibiotics in low resource settings. Health care workers prefer broad spectrum prescribing if there is a high level of diagnostic uncertainty in absence of bacteriological culture and local AMR data [48–50]. Fear of bad treatment outcomes of critical diseases together with inadequate time and attitude of critical analysis also contribute to the prescription of multiple and broad spectrum antimicrobials [38, 51]. Poor knowledge about AMR and antibiotic use - recently demonstrated among of final year medical and pharmacy students in East Africa [52] - probably adds to this practice. Senior medical prescribers act as key opinion leaders which influence the prescribing trend of their juniors resulting in a cascading effect on prescription practices [38]. Further, patient demands for “strong” antibiotics [47], personal familiarity with certain FDC antibiotic brands and promotional practices of pharmaceutical industry have their influence [51, 53–55].

Access to antibiotics and market mechanisms play a role too [53, 56]. An example is the non-availability of cloxacillin (first choice treatment for infections with susceptible *S. aureus*) as a single component in India – resulting in the detrimental use of FDCs containing cloxacillin [57] – an observation which was made by one of the authors in Tanzania (KVJ) too. Availability can also be influenced by costs and pharmaceutical interests on national or institutional level [1, 56, 58]. We found no aggregated data on availability or costs of non-recommended FDCs compared to preferable single ingredient alternatives.

The development of a new FDC is often driven by economic arguments: by combining different drugs without patent, one could establish a patentable medicine to generate profit [58]. Reformulating individual ingredients into a new FDC makes it possible to evade price control [38]. The medicine market in India abounds in branded generics, which favors brand loyalty and is a strong market mechanism [56]. Of note, use of WHOCC ATC classification use incorrectly may give the impression that the FDC antibiotics listed were more appropriate than being non-listed [59].

### Regulation and international trade

India has the highest number of antimicrobial FDC antibiotics available in the market [1]. Efforts by the Indian Central Drugs Standard Control Organization (CDSCO) to ban FDC antibiotics in 2016 have been challenged by the pharmaceutical industry, resulting in long-lasting legal battles [37]. Although the Indian Government banned 26 FDC antibiotics in 2018, these banned products are partially still available in the Indian market or were replaced by comparable alternatives after minor adjustments. This indicates a need for stricter implementation of the regulatory decision [1, 58].

FDC antibiotics that have been banned in India are exported to other African and Asian countries. The export is deemed legal if the importing country has no objections [60, 61]. A review on FDC antibiotics in Nigeria showed that one third of FDC medicine -among which a large share of antibiotics- was produced in India, many of which were not listed on the Nigerian nor the WHO EML and classified as Not Recommended [62]. In Uganda, ampicillin-cloxacillin was removed from the 2016 Uganda Clinical Guidelines, nevertheless it was still widely prescribed in the years 2020–2021 based on a point prevalence survey on antimicrobial use in 13 Ugandan hospitals [63]. Reasons for its use could not be elucidated.

In Tanzania, lack of access to antibiotics still results in a considerable death toll for example among children with respiratory tract infections [64]. As a consequence it would not be feasible for the TMDA to restrict antibiotic use to prescriptions by a medical doctor only. The proportion of private-sector antibiotic consumption in Tanzania is increasing annually, while the local drug stores are not obliged to comply with the Tanzanian STG [24]. Globally a considerable amount of antimicrobial consumption takes place in the private sector, including informal ones which are difficult to control [24, 56, 65].

### How to counter the use of not-recommended FDC antibiotics?

First, there is an urgent need for data on antibiotic consumption, export and pharmaceutical sales including non-recommended FDC-AB, with a focus on lower- and middle-income countries. The most recent published data describe the years 2000–2015 and Sub-Saharan African countries are seriously underrepresented (16–17). The recently published MAAP policy brief is an encouraging initiative, with an international governance structure involving both local scientists (ASLM) as well as international stakeholders like the Africa CDC, the One Health Trust, the African Union Heads of State and Government, West African Health Organization (WAHO) and ECSA-HC (East, Central and Southern Africa Health Community) [69]. This framework, supported by the Fleming fund, will help build the first two strategic objectives of the AMR action plan: creating awareness and strengthening the knowledge and evidence base [70]. WHO provides a technical basis for setting up national surveillance systems on antimicrobial consumption (AMC), as a component of the Global Antimicrobial Resistance and Use Surveillance System (GLASS) [67]. We underline that these real-time data on antimicrobial use are pivotal to visualize the magnitude of use and likewise detrimental export of non-recommended FDC-AB to low- and middle-income countries.

Second - to overcome the gap between science and practice- it is essential to investigate the contribution of the determinants related to use of the non-recommended FDC-AB in low- and middle-income countries. They can be translated into improvement opportunities: improving national guidelines, creating awareness among prescriber and patients, diagnostic capacity building, availability of local and national surveillance data, development of an evidence based national antibiotic formulary, monitor and censor misleading promotional practices, assuring a healthy market of accessible single ingredient antibiotics, and strengthening national as well as international regulations on import and distribution of antibiotics [1, 51, 66]. The MAAP project calls for regulations to remove antibiotics not categorized in WHO’s AWaRe system from the National Essential Medicine Lists and enforce the interdiction of unclassified antibiotics, including non-recommended fixed-dose combinations [69]. This initiative as well as the diverse list of determinants above indicate that antimicrobial stewardship is only possible in a supranational multisectoral approach.

Ensuring adequate funding for implementation in the field is essential to the success of interventions aimed at containing AMR, particularly in low- and middle-income countries where the disease burden is high. Although the financial costs and health outcomes associated with AMR interventions, such as the elimination of non-recommended fixed-dose combination antibiotics (FDC-AB), have not been quantified, various investments are necessary for antimicrobial stewardship. These investments include human capital, diagnostic infrastructure (including consumables), access to alternative drugs, and public awareness [70, 71]. Therefore, there is a pressing need to establish a business case for AMR interventions in low-resource settings such as Tanzania [69, 70].

## Conclusion

Based on the example of Tanzania, we discussed the lack of microbiological, pharmacological and clinical evidence for non-recommended antibiotic fixed drug combinations (FDC-AB) as well as the concerns of appropriate dosing, safety and risk for antimicrobial resistance (AMR). Given their low regulatory capacity, low- and middle-income countries – most hit by the worldwide AMR- are vulnerable to the supply of non-recommended FDC-AB when searching for accessible, affordable antibiotics (16–17, 51). Contributing factors are lack of diagnostics, poor training in antibiotic prescribing, patients’ preferences, role-model of senior prescribers and pharmaceutical promotion. International market mechanisms include economic motivation for development, branding and promotion, poor access to the single antibiotic forms and weak national regulatory capacity [1, 51, 66].

Data on antimicrobial use are pivotal to visualize the magnitude of use and likewise detrimental export of non-recommended FDC-AB to low- and middle-income countries [16, 17, 69]. Developing a business case for AMR interventions in low and middle income countries is a topic in need of exploration [69, 70]. A supernational multisectoral approach is needed in order to abolish availability and use of non-recommended FDC-AB which conflict with antibiotic stewardship efforts in low-resource settings like Tanzania.

## Electronic supplementary material

Below is the link to the electronic supplementary material.

Supplementary Material 1

## Data Availability

Not applicable.

## List of abbreviations

AMC: Antimicrobial consumption
AMR: Antimicrobial resistance
ATC: Anatomical Therapeutic Chemical (ATC) classification system (of WHO)
AWaRe: Access, Watch and Reserve (WHO antibiotic classification)
BL-BLI: Beta-lactam antibiotic with beta-lactamase inhibitor
CDSCO: Central Drugs Standard Control Organization (of India)
DDD: Defined Daily Doses
EMA: European Medicines Agency
EML: Model List of Essential Medicines
EUCAST: European Committee on Antimicrobial Susceptibility Testing
FDC: Fixed-dose combinations
GLASS: Global AMR and Use Surveillance System (of WHO)
FDC-AB: Antibiotic fixed-dose combinations
MAAP: Mapping Antimicrobial Resistance and Antimicrobial Use Partnership
MIC: Minimal Inhibitory Concentration
NEMLIT: National Essential Medicine List (of Tanzania)
LMIC: Low- and Middle-Income Countries
STG: Standard Treatment Guidelines (of Tanzania)
TMDA: Tanzanian Medicines and Medical Devices Authority
WHO: World Health Organization
WHOCC: WHO Collaborating Centre of Drugs Statistics Methodology
