# Supplementary material for: Not recommended fixed-dose antibiotic combinations in low- and middle-income countries – the example of Tanzania
Source: Antimicrob Resist Infect Control. 2023 Apr 19;12:37. doi: 10.1186/s13756-023-01238-8 (PMC10116708; doi:10.1186/s13756-023-01238-8)
Supplement: Supplementary file 1 — Supplementary Material 1 [file 13756_2023_1238_MOESM1_ESM.docx]

| **Supplementary Table 1: Approved FDC antibiotics by WHO AWaRe classification, WHO EML list, FDA and EMA** | | | | |
| --- | --- | --- | --- | --- |
| **Drug combination** | **WHO AWaRe classification (11,12)** | **WHO EML (14)** | **FDA approval (9)** | **EMA approval (13)** |
| Amoxicillin-clavulanic acid | Access | Yes | Yes, 2002. Only letter. | European Commission final decision. Need for harmonization. |
| Ampicillin-sulbactam | Access^1^ | No | Yes, 2002. Only letter available, no label or review. | European Commission final decision. Need for harmonization. |
| Ceftazidime-avibactam | Reserve | Yes | Yes, 2015 | Authorized, 2016 |
| Ceftolozane-tazobactam | Reserve^1^ | No | Yes, 2014 | Authorized, 2015 |
| Dalfopristin-quinupristin | Reserve^1^ | No | Yes, 1999 | Not listed |
| Imipenem-cilastatin-relebactam | Reserve^1^ | No | Yes, 2019 | Authorized, 2020 |
| Meropenem-vaborbactam | Reserve | Yes | Yes, 2017 | Authorized 2018 |
| Piperacillin-tazobactam | Watch | Yes | Yes, 2010. No label available. | European Commission final decision. Need for harmonization. |
| Sulfadiazine-tetroxoprim | Access^1^ | No | Not listed | Not listed |
| Sulfadiazine-trimethoprim | Access^1^ | No | Not listed | Only listed for veterinary use |
| Sulfadimidine-trimethoprim | Access^1^ | No | Not listed | Not listed |
| Sulfamerazine-trimethoprim | Access^1^ | No | Not listed | Not listed |
| Sulfamethizole-trimethoprim^3^ | Access^1^ | No | Not listed | Not listed |
| Sulfamethoxazole-trimethoprim | Access | Yes | Yes, 1986. No label | Not listed or nationally authorized |
| Sulfametrole-trimethoprim | Access^1^ | No | Not listed | Not listed |
| Cefcapene-pivoxil^2^ | Watch^2^ | No | Not listed | Not listed |
| Cefditoren-pivoxil^2^ | Watch^2^ | No | Yes, 2001. Marketing: discontinued | Not listed |
| Cefetamet-pivoxil^2^ | Watch^2^ | No | No | Not listed |
| Cefpodoxime-proxetil^2^ | Watch^2^ | No | Yes, 2007. No label | Not listed |
| Ceftaroline-fosamil^2^ | Reserve^2^ | No | Yes, 2021. Marketing: discontinued | Authorized, 2012 |
| Cefteram-pivoxil^2^ | Watch^2^ | No | Not listed | Not listed |
| Ceftobiprole-medocaril^2^ | Reserve^2^ | No | Not listed | Refused, 2010 |
| Imipenem-cilastatin^2^ | Watch^2^ | No | Yes, 2011 | European Commission final decision. Need for harmonization. |
| Sulfamoxole-trimethoprim^2^ | Access^2^ | No | Not listed | Not listed |
| Note: Combinations of antibiotics intended for treatment or prophylaxis of tuberculosis or malaria are excluded from this overview  ^1^ WHO AWaRe classification states this FDC was listed for monitoring purposes (11)  ^2^ Listed only in the Excel database (11), but not the online AWaRe database (12)  ^3^ Sulfamethizole + trimethoprim was only listed online (12), but not in the Excel AWaRe classification (11)  Abbreviations :  FDC: Fixed-dose combination WHO: World Health Organization AWaRe: Access, Watch, Reserve classification  EML: Model List of Essential Medicines  FDA: Food and Drug Administration  EMA: European Medicines Agency | | | | |
